# The Influence of IL-11 on Cardiac Fibrosis in Experimental Models: A Systematic Review

**DOI:** 10.3390/jcdd11020065

**Published:** 2024-02-17

**Authors:** Yarlla Loyane Lira Braga, José Rodrigues do Carmo Neto, Pablo Igor Ribeiro Franco, Fernanda Rodrigues Helmo, Marlene Antônia dos Reis, Flávia Aparecida de Oliveira, Mara Rúbia Nunes Celes, Marcos Vinícius da Silva, Juliana Reis Machado

**Affiliations:** 1 Department of Bioscience and Technology, Institute of Tropical Pathology and Public Health, Federal University of Goias, Goiania 74605-450, GO, Brazil; yarlla_lira@hotmail.com (Y.L.L.B.); rodriguesnneto@gmail.com (J.R.d.C.N.); pablo_igor@hotmail.com (P.I.R.F.); flavia@ufg.br (F.A.d.O.); mrubia_celes@ufg.br (M.R.N.C.); 2 General Pathology, Federal University of Triângulo Mineiro, Uberaba 38025-180, MG, Brazil; fernandahelmo@gmail.com (F.R.H.); mareispatologia@gmail.com (M.A.d.R.); 3 Department of Microbiology, Immunology and Parasitology, Institute of Biological and Natural Sciences, Federal University of Triângulo Mineiro, Uberaba 38025-180, MG, Brazil; marcos.silva@uftm.edu.br; 4 Department of General Pathology, Federal University of Triângulo Mineiro, Uberaba 38025-180, MG, Brazil

**Keywords:** interleukin-11, fibrosis, ERK1-2 pathway, IL-11 receptors, heart diseases

## Abstract

Fibrosis is one of the main factors that impair the function of many organs. In the heart, fibrosis leads to contractile dysfunction and arrhythmias, which are important in the development of heart failure. Interleukin (IL)-11 is regulated in various heart diseases and has recently been reported to be an important cytokine in fibrosis in this organ. However, this topic has been little explored, and many questions persist. Thus, this systematic review aimed to report on possible IL-11 therapies evaluated in rodent model-induced cardiac fibrosis. Inclusion criteria were experimental in vivo studies that used different rodent models for cardiac fibrosis associated with IL-11 interventions, without year and language restrictions. The search in PubMed, Web of Science, and Embase databases was performed in October 2022. The risk of bias assessment of the studies was based on the guidelines of the SYRCLE tool, and data from the selected articles were also presented in a table as a narrative description. This review was based on eight studies in which five different interventions were used: recombinant human IL-11 (rhIL-11), anti-IL11 (X203), recombinant mouse IL-11 (rmIL-11), lentivirus (LV)-IL-11 + lutein, and anti-IL11RA (X209). Based on the included studies, the results were variable, with IL-11 overexpression inducing cardiac fibrosis, while inhibition protected against this process, preserving the function of this organ. Therefore, IL-11 stands out as a promising therapeutic target for cardiac fibrosis. However, further studies are needed to understand the mechanisms triggered by each treatment, as well as its safety and immunogenicity.

## 1. Introduction

Fibrosis that occurs in response to tissue injury is one of the main factors that compromises the function of several organs, which is coordinated by oxidative stress, inflammation, apoptosis, and numerous signaling pathways. Cardiac injury can be induced by a variety of elements, including myocardial infarction, hypertension, valve disease, and myocarditis, and promotes tissue remodeling which is important in the development of heart failure (HF) [1,2]. HF affects approximately 40 million people worldwide and is a leading cause of morbidity, hospitalizations, poor quality of life, and reported death. Projections through 2030 in the United States alone suggest a total HF treatment spending of $69.7 billion [3,4,5].

Recently, IL-11 was found to be an important factor in fibrosis in the kidney [6,7,8], liver [9,10,11], lung [12,13,14,15], and heart [8,15,16,17,18]. It is a cytokine member of the IL-6 cytokine family that requires activation of the gp130 receptor for its cellular activity [19]. Associated with the gp130 receptor, the IL-11Rα receptor receives the signal from this cytokine and triggers the activation of several transcription factors, including the signal transducer and activator of transcription proteins (STAT) and the extracellular signal-regulated kinase (ERK) pathway [20,21]; the latter is an important pathway for IL-11-induced fibrosis in the heart [15,22]. In humans, serum IL-11 is increased in patients with chronic heart failure [17], coronary heart disease [23], and aortic dissection [24]. Cardiac IL-11 expression in a murine model is elevated in ischemia/reperfusion-induced myocardial infarction [25], cardiac fibrosis induced by angiotensin II (Ang II) [5,26], transverse aortic constriction (TAC) [26], and ascending aortic constriction (AAC) [27]. Studies in knockout mice for the IL-11Rα receptor demonstrated protection against fibrosis [15] while IL-11 treatment in the myocardial infarction model induced decreased cardiac fibrosis [25].

Different IL-11-based therapies have been developed and investigated for the assessment of fibrosis in various organs [6,8,27,28,29]. However, currently, whether this therapy can effectively treat cardiac fibrosis remains controversial. Mainly because the first studies used rhIL-11 in murine model, and it was later discovered recombinant IL-11 is species-specific and that this treatment works as an inhibitor of this cytokine in mice. In this context, this systematic review aimed to evaluate the influence of IL-11 treatment as a possible stimulator or inhibitor of cardiac remodeling in a rodent model of cardiac fibrosis.

## 2. Materials and Methods

### 2.1. Search Strategy

The systematic review was conducted per the methodological guidelines proposed by the Main Items for Reporting Systematic Reviews and Meta-Analyses (PRISMA) [30]. The protocol of this review was registered in the PROSPERO (International Prospective Register of Systematic Reviews) database, under registration number CRD42022350693. For execution of the literature search, three search bases were used: PubMed, Web of Science, and Embase, with the keywords IL-11, Heart, Myocardium, Fibrosis, and Cicatrization. (https://www.crd.york.ac.uk/prospero/display_record.php?ID=CRD42022350693 accessed on 2 September 2022)

### 2.2. Inclusion/Exclusion Criteria

The systematic review in question was based on the question: “Does IL-11 have any influence on cardiac fibrosis?”. Thus, to assemble the search strategy and establish eligibility criteria, we used the acronym model PICOT (population, intervention, comparator, outcome, and types of studies); P: rodent models of cardiac fibrosis; I: treatment intervention; C: no treatment (control group); O: cardiac remodeling; T: in vivo studies. Therefore, only experimental in vivo studies on knockout animals using different experimental methodologies for cardiac fibrosis with treatment interventions for IL-11 were included. For the exclusion criteria, the following points were followed: (1) not murine models of cardiac fibrosis or murine with genetic modifications, in vitro, ex vivo, in silico, and human studies; (2) not pharmacological treatment with IL-11 or any IL-11 inhibitor; (3) studies that focus on treatment and do not assess cardiac tissue; (4) studies that do not compare treated animals with untreated animals; and (5) any measurement that does not show a biological effect.

### 2.3. Data Extraction and Risk of Bias Assessment

The first step in the selection of articles was performed by two reviewers (Y.L.L. B and J.R.C. N) independently and blinded to the author. To assist in this step, the Rayyan—Intelligent Systematic Review (https://www.rayyan.ai/ accessed on 6 September 2022) program was used. The data extracted from the selected studies comprised intervention, treatment schedule, and concentration of/treatment route, experimental model, fibrosis model, the form of induction of cardiac fibrosis, time of euthanasia, organ and region evaluated, methodology used for fibrosis analysis/analyzed, region fibrosis, IL-11 cardiac levels, cell death, cardiac function, fibrosis markers, mortality rate %, body weight, immunomodulatory effects, fibrosis pathway, and references (Table 1).

To assess the risk of bias, we used the Systematic Review Center for Laboratory Animal Experimentation (SYRCLE) tool [31]. This step was also performed by two reviewers independently (Y.L.L.B and P.I.R.F). The tool consists of six categories: selection bias, performance bias, detection bias, attrition bias, reporting bias, and other sources of bias.

**Table 1:** The data extracted from the selected studies.

Intervention	Treatment Schedule	Intervention Concentration of/Treatment route	Experimental Model	Fibrosis Model	Form of Induction of Cardiac Fibrosis	Euthanasia	Organ and Region Evaluated	Methodology Used for Fibrosis Analysis	Fibrosis	IL-11 Cardiac Levels	Cell Death	Cardiac Function	Fibrosis Markers	Mortality rate %	Body Weight	Immunomodulatory Effects	Fibrosis Pathway	Reference
Anti-IL11 (X203) Anti-IL11RA (X209)	24 h post-surgery biweekly for 2 weeks	20 mg/kg intraperitoneal	Male mice C57BL/6J (10- to 12-week-old)	Cardiac fibrosis	Ascending aortic constriction (AAC)	2 weeks post-AAC	-	Masson’s trichrome	X203 and X209 decrease cardiac and perivascular fibrosis	X209 reduced IL11	-	X203 and X209: increased aortic flow velocities and pressure gradients X209: decreased aortic root diameter. HW/BW rate not changed	X203 and X209 decrease. RNA expression: Col1a1, Col1a2, Col3a1, Fn1, Mmp2 and Timp1 X203 and X209 decrease. Protein expression: collagen and collagen III	Not changed	Not changed	-	X203 and X209 not changed: SMAD2, SMAD3. STAT3 X203, and X209 decreased ERK1/2	[27]
Anti-IL11 (X203)	24 h post- surgery biweekly for 2 weeks or 4 weeks	20 mg/kg^−1^ intraperitoneal	Male mice C57BL/6J (10–12 weeks old)	Cardiac fibrosis	Transverse aortic constriction (TAC) AngII-subcutaneous	2 weeks 4 weeks	Left ventricular	Masson’s trichrome	X203 decrease cardiac and perivascular fibrosis	X203 not changed IL11 levels	-	Not changed	X203 decrease Col1a2, Col3a, and FN1	Not changed	-	-	-	[26]
Lentivirus (LV)-IL-11 + lutein	LV-IL-11: Two weeks before Ang II	LV-IL-11: Five separate intramyocardial injections (5 μL) into the front, side, and back of the left ventricle.	-	Cardiac fibrosis	AngII	Chronic	Left ventricular	Picrosirius red	lutein + LV-IL-11 increase	-	-	lutein + LV-IL-11 increased HW/BW, LVEDD and decreased EF	lutein + LV-IL-11 increased expression of ROS, Col1, TGF-β1 NOX2, and NOX4	-	-	-	-	[5]
Recombinant human IL-11 (rhIL-11)	10 min before the heart was isolated for cold ischemia	12 µg/kg intravenously	Male Sprague- Dawley rats	Myocardial infarction	Ischemia/reperfusion	-	Left ventricular	-	-	-	Recombinant human IL-11 decrease apoptotic cells	Recombinant human IL-11 recovery of cardiac contractile function with increased LVDP and ±dP/dt	-	-	-	-	-	[32]
Recombinant mouse IL-11 (rmIL-11)	Daily for 6 successive days.	Dose not found. Subcutaneous	Male mice C57BL/6 (10–12 weeks)	Myocardial infarction	Ischemia/reperfusion	-	-	Masson’s trichrome	Recombinant mouse IL-11 increase	-	-	Recombinant mouse IL-11 increased end-systolic volume, epicardial thickness, and decreased ejection fraction (%)	-	-	-	-	-	[15]
Recombinant human IL-11 (rhIL-11)	At start of reperfusion	3, 8, 20, 50 µg/kg intravenously	C57BL/6 e (8 to 12 week old)	Myocardial infarction	Ischemia/reperfusion	Twenty-four hours after reperfusion	Left ventricular	-	-	-	20 µg/kg: decreased apoptotic cells	20 and 50 µg/kg: decreased risk area size and infarct size 20 µg/kg; increased ejection fraction, fractional shortening, and hemodynamics	20 µg/kg: increased MT1, MT2, and decreased ROS	Not changed	-	-	20 µg/kg: increased STAT3	[18]
Recombinant human IL-11 (rhIL-11)	24 h after MI operation for 5 days consecutively	3 µg/kg, 8 µg/kg, and 20 µg/kg intravenously	C57BL/6 (10 weeks old)	Myocardial infarction	Ischemia/reperfusion	14 days after MI	Left ventricular	Masson’s trichrome	Both fibrotic circumference and fibrotic area were reduced by IL-11 in a dose-dependent manner	-	Decrease apoptotic cells	IL-11 treatment ameliorated cardiac dysfunction in a dose-dependent manner	-	-	-	-	IL-11 treatment increase STAT3 in a dose-dependent manner	[25]
Recombinant human IL-11 (rhIL-11)	15 h before the ischemia/reperfusion injury	8 µg/kg intravenously	C57Bl/6 (10 weeks old)	Myocardial infarction	Ischemia/reperfusion	14 days after MI	-	-	-	-	-	Recombinant human IL-11 decreased risk area size and infarct size	-	-	-	-	-	[33]

## 3. Results

### 3.1. Search for Studies and PRISMA Flowchart

**Figure 1:**
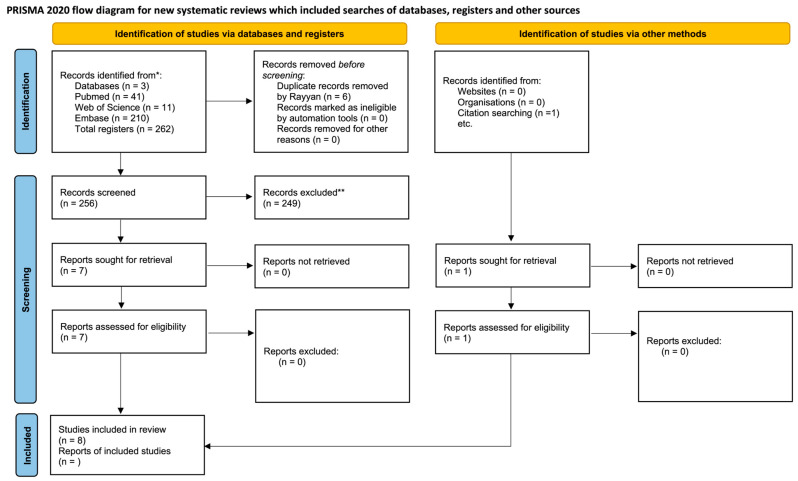
PRISMA (Preferred Reporting Items for Systematic Reviews and Metanalyses) flow chart of the study selection and inclusion process [30].

The initial database search generated 226 records, with 41, 11, and 210 found in PubMed, Web of Science, and Embase, respectively. In the duplicate article screening step, 6 articles were excluded, with a total of 256 retained for the analysis. Next, analysis by title and abstract resulted in the inclusion of seven potential articles and the exclusion of two hundred and forty nine. Through a manual search in the reference list, one more article was selected, totaling eight. Thus, eight articles were considered eligible and subjected to qualitative analyses (Figure 1).

### 3.2. Characteristics of the Studies

The first publication focusing on IL-11 intervention for in vivo cardiac fibrosis analysis was reported in 2007. Thereafter, 2017 and 2021 were the years with the highest number of publications (2), representing 50% of the total articles during this period. The year 2021 was also the period of the last publication on the topic. 

To conduct the experiments, only two species were used as models: *Mus musculus* (C57Bl/6) and *Rattus norvegicus* (Sprague-Dawley). The former was the most commonly used in seven studies. Five different interventions were used in the included articles: recombinant human IL-11 (rhIL-11), anti-IL11 (X203), recombinant mouse IL-11 (rmIL-11), lentivirus (LV)-IL-11 + lutein, and anti-IL11RA (X209). Among these, rhIL-11 was the most commonly used (four articles), followed by X203 (two articles).

Myocardial infarction and cardiac fibrosis were the models used for cardiac analysis, totaling five and three articles, respectively. Such models were induced in four different ways: ascending aortic constriction (AAC), transverse aortic constriction (TAC), angiotensin II, and ischemia/reperfusion. Ischemia/reperfusion was the most reported method (five articles), followed by Ang II (two articles); and TAC and AAC (one each).

### 3.3. Risk of Bias Assessment

**Figure 2:**
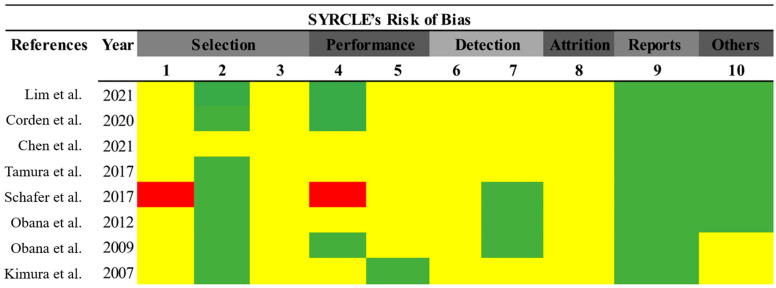
Risk of bias assessment. Prepared using the SYRCLE tool [31]. Green (low risk of bias); red (high risk of bias); yellow (uncertain risk of bias) [5,15,18,25,26,27,32,32].

To evaluate the risk of bias, all eight included articles were analyzed. As shown in Figure 2, most articles did not clearly address the criteria for selection bias (items 1–3), performance (item 5), detection (items 6 and 7), and attrition (item 8). Only one of the selected articles presented a risk of bias. Finally, 75% of the selected papers did not report information regarding a possible conflict of interest.

### 3.4. Relationship between IL-11 and Cardiac Fibrosis

For the studies evaluating the relationship between IL-11 and cardiac fibrosis, Masson’s trichrome or picrosirius red were the stains used (five studies). Masson’s trichrome method was preferred for collagen analysis (80%), and only one study (20%) used picrosirius red.

The levels of cardiac fibrosis were analyzed in five articles. IL-11 inhibition by treatment with anti-IL11 (X203) or anti-IL11RA (X209) induced a decrease in cardiac and perivascular fibrosis levels [26,27]. A similar result was demonstrated by the rhIL-11 treatment, but in a dose-dependent manner [25]. Refuting these studies, increased cardiac fibrosis was stimulated by treatment with LV-IL-11+lutein [5] and rmIL-11 [15].

### 3.5. Relation between IL-11 and Fibrosis Markers

To analyze the effect of IL-11 on fibrosis induction, different cardiac remodeling markers were quantified using various techniques. In this context, only four of the eight selected studies evaluated the relationship between IL-11 and fibrosis markers (Table 1). Treatment with X203 or X209 reduced the levels of production of collagen 1 alpha 1 and 2 (COL1A1 and COL1A2), collagen 3 alpha 1 (COL3A1), fibronectin 1 (FN1), matrix metalloproteinase-2 (MMP2), and tissue inhibitor of metalloproteinase-1 (TIMP1) [26,27]. In these studies, it was observed that only treatment with X209 was able to reduce cardiac levels of IL11 and IL11RA. One selected study used an association of LV-IL-11 with lutein for treatment and thus an increase in the levels of reactive oxygen species (ROS), collagen I (COL1), transforming growth factor (TGF-β1), NADPH oxidases 1 and 2 (NOX2 and NOX4) [5]. For rhIL-11, treatment increased the levels of metallothionein 1 and 2 (MT1 and MT2) and decreased ROS [18].

### 3.6. Relationship between IL-11/Fibrosis Pathways

Among the eight studies selected for this systematic review, the relationship between IL-11 and fibrosis pathways was evaluated in only three studies. The analyzed pathways were STAT3 (two articles), SMAD2 and SMAD3 (one article), and ERK 1/2 (one article) (Table 1). Among the studies evaluated, the STAT3 pathway was predominantly induced by rhIL-11 treatment; this result was obtained in a dose-dependent manner [25] and another by the treatment dose of 20 µg/kg [18]. SMAD2, SMAD3, signal transducer, and activator of transcription proteins (STAT3) production was not altered by treatment with X203 or X209; however, the latter treatment decreased ERK1/2 production [27].

### 3.7. Relation between IL-11 and Cardiac Function

Cardiac performance was evaluated based on aortic parameters, including aortic flow velocity, pressure gradient, aortic root diameter, and cardiac parameters, including cardiac wall thickness, ejection fraction (EF), maximum left ventricular developed pressure (LVDP), peak rates of positive and negative changes in left ventricular pressure (±dP/dt), end-systolic volume, left ventricular end-diastolic diameter (LVEDd), infarct area size, hemodynamics, and relative heart weight (HW/BW).

All the selected studies evaluated the cardiac function influenced by the different treatments associated with IL-11 (Table 1). For mice in which cardiac fibrosis was induced by the AAC method, treatment with X209 or X203 was able to benefit the model by inducing an increase in aortic flow velocity and pressure gradient and a decrease in aortic root diameter. Neither treatment changed the cardiac parameters [27]. Similarly, treatment with X203 did not induce changes in cardiac function in another included study either [26]. In the same AngII-induced cardiac fibrosis model, the association of LV-IL-11+lutein manifested a significant increase in the HW/BW ratio and LVEDd, and a significant decrease in EF [5].

For rhIL-11 treatment in ischemia/reperfusion-induced myocardial infarction models, the effects of IL-11 improved cardiac function, with increased LVDP, ±dP/ [25,32], and reduction in the size of the infarct area [18,33]. In addition, treatment with the 20 µg/kg dose increased hemodynamics and EF [18]. In contrast, for the same model, but with rmIL-11 intervention, overexpression of this cytokine increased end-systolic volume and epicardial thickness and decreased EF [15].

Another factor also associated with the evolution of the cardiac fibrosis process is cell death. For this, cardiomyocyte apoptosis data were collected from the selected studies. The influence of IL-11 on this process was demonstrated in three articles. Only rhIL-11 treatment reported the influence of IL-11 on apoptosis (Table 1); overall, the intervention decreased cardiomyocyte apoptosis in infarction models [18,25,32]

### 3.8. Relationship between Treatment and General Parameters of the Experimental Model

The clinical aspects of experimental models are relevant parameters for the analysis of the interventions applied. Among the selected studies, only four studies have focused on these parameters. In this case, 75% evaluated the mortality rate, while only one reported body weight (25%). Therefore, treatment with X203 or X209 [26,27] and rhIL-11 [18] did not cause any change in the clinical aspects of the experimental models evaluated.

## 4. Discussion

**Figure 3:**
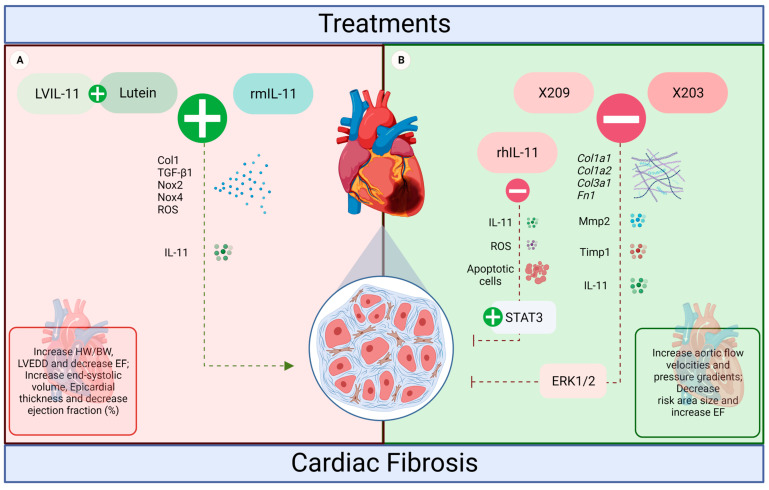
The influence of IL-11 treatments on the heart of rodents. (**A**) LVIL-11 + lutein and rmIL-11 induced a pro-fibrotic action in the heart (red, worst prognosis). Both treatments potentiated the effects of IL-11, increasing the levels of ROS, Col1, TGF-β1, NOX2, and NOX4, which resulted in increased fibrosis and impaired cardiac function. (**B**) anti-IL-11 antibodies X203 and X209, and rhIL-11 inhibited cardiac fibrosis (green, beneficial effects). For the inhibitors, the effects were a reduction in IL-11 expression levels and fibrosis associated with the protection of cardiac function. Both antibodies also reduced Col1a1, Col1a2, Col3a1, Fn1, Mmp2, Timp1, and ERK signaling. While rhIL-11 reduced cardiomyocyte apoptosis, ROS increased STAT3 (created with BioRender.com accessed on 2 September 2022).

Based on different interventions of the cytokine IL-11, the present study summarizes its influence on an in vivo cardiac fibrosis model. In this context, to date, five different IL-11 treatments have been explored in the literature: recombinant human IL-11, recombinant mouse IL-11, lentivirus (LV)-IL-11 + lutein, anti-IL11 (X203) and anti-IL11RA (X209). However, some controversies about the relationship of IL-11, fibrosis, and the signaling pathways of this process in cardiac pathologies have not yet been fully resolved (Figure 3).

Due to its initial characterization as a hematopoietic cytokine, the first described IL-11 treatment was rh-IL11, used to reduce thrombocytopenia in chemotherapy patients [34]. However, its initial use in rodents has led to a misunderstanding as to IL-11′s true biological function. Recombinant IL-11 is species-specific, which, in murine fibroblasts, in vitro, rhIL-11 does not induce a biological effect while in mice, it can act as an inhibitor of endogenous IL-11 [29]. Therefore, the first reports of rhIL-11 treatment in rodents induced cardiac tissue protection associated with IL-11 inhibition which resulted in an antifibrotic effect [18,25,32,33].

Indeed, the articles included in this systematic review showed that inhibition of IL-11 with anti-IL11 (X203) or anti-IL11RA (X209) antibodies in a cardiac fibrosis model resulted in a reduction of COL1A, COL1A2, COL3A1, FN1, MMP2, and TIMP1, fibrosis and a reduction in ERK1/2 in the heart [26,27]. The antibodies X203 and X209 were recently developed and effectively blocked the fibrotic response in mice, and are used for preclinical testing in murine model. Corroborating the cardiac findings, in a model of nonalcoholic steatohepatitis, IL-11 inhibition by X203 or X209 decreased liver fibrosis by suppressing ERK activation and consequently MMP2 and TIMP1 [35]. Similar results were observed in arterial remodeling [36], Marfan syndrome [28], and pulmonary fibrosis, which shows the influence of IL-11 on fibrosis [37].

The treatment with IL-11 recombinant specie-specific, rmIL-11, in the model of myocardial infarction, induced an increase in cardiac fibrosis associated with left ventricular impairment [15]. Besides the cardiac effects cited, in other studies, this treatment significantly increased fibrosis levels in models of pulmonary fibrosis [37,38], nonalcoholic steatohepatitis [35], renal models [15], and vascular remodeling [39]. In general, treatment with rmIL-11 induces profibrotic mechanisms by stimulating ERK1/2, as demonstrated in vitro and in vivo [27,35,39]. These results highlight the importance of the IL-11/ERK pathway in fibrotic signaling in the heart.

IL-11 treatment for cardiac fibrosis was also demonstrated using lentivirus (LV)-IL-11 developed from plasmids. Administration was by intramyocardial injections and subsequently associated with lutein treatment. Mice with induced cardiac fibrosis that received (LV)-IL-11 + lutein exhibited increased cardiac TGF-β levels, concomitant with other markers such as ROS, COL1, NOX2 and NOX4, and cardiac fibrosis. Lutein is a natural dihydroxycarotenoid that can be obtained in fruits and vegetables, and its use is favorable for rodent models of cardiac fibrosis by reducing this pathological process by inhibiting the AP-1/IL-11/ERK pathway. AP-1 transactivates the IL-11 promoter by binding to cis-elements to increase IL-11 expression [40]. Thus, it was shown that IL-11 overexpression induced by (LV)-IL-11 predominated and weakened the protective effect of lutein when these treatments were associated [5]. In this context, the IL-11/ERK signal seems to be the main mediator of fibroblast to myofibroblast transformation, besides acting downstream of TGF-β and increasing the production of profibrotic factors [15,37,41,42,43]. ERK signaling is important for the profibrotic effects of IL-11 since its phosphorylation drives the expression of factors such as ACAT2, COL1A, COL1A2, COL3A1, FN1, MMP2, and TIMP1 [13,15,27,29,37]. Effects that were not produced by IL-11-dependent phosphorylation in other pathways, such as SMAD3, STAT1/3 [15,27,44,45].

Cell death and inflammatory response in the face of pathological processes are also parameters that influence cardiac dysfunction and remodeling; however, few studies have reported and explored the relationship between these aspects and IL-11 in models of cardiac fibrosis in vivo. Recently, it was shown that IL-11 expression in murine cardiomyocytes was associated with up-regulation of cytokines, chemokines, complement factors and an increase in inflammatory cells [46]. The treatment with rmIL-11 in mice showed that activation of the inflammatory response in the heart is associated with activation of the STAT3 pathway [47]. However, further investigations are needed to elucidate these effects more clearly for a better understanding of the biological role of IL-11 in cardiac fibrosis.

Despite the methodological diversity among the studies for the induction of cardiac fibrosis (route of administration, concentration of the intervention, and experimental design), this variation did not interfere with the model because all the studies obtained results according to the proposed objective. In addition, because this is a recent topic, studies are scarce in the literature, and for some treatments, it was not possible to perform the model comparison. In addition, most of the included studies did not clearly report all the items evaluated using the SYRCLE tool, thus making a complete analysis of the methodological quality impossible. To circumvent this limitation at the study level, the authors of future studies should describe in more detail the methodology of the study to ensure better reproducibility and reliability. Moreover, it is noteworthy that due to the heterogeneity of the studies included, meta-analysis was not tested, which is a limitation at the level of this systematic review. On the other hand, a comprehensive search, including a Latin American database, was performed to find all articles that fit the guiding theme.

## 5. Conclusions

The present systematic review involved studies that tested possible IL-11 therapies for the treatment of cardiac fibrosis in rodent models. Thus, five different types of treatments were described: rhIL-11, rmIL-11, LV-IL-11 + lutein, X203, and X209, which showed different modes of action. Therefore, the rhIL-11 and antibodies X203 and X209 inhibited the effects of IL-11 and showed anti-fibrotic activity, resulting in cardiac protection associated with a reduction in pro-fibrotic factors and improved cardiac performance. However, treatment with rmIL-11 intensified the effects of IL-11 and thus induced increased fibrosis, compromising cardiac function. Similarly, the association of LV-IL-11 + lutein as treatment predominated the effects of IL-11, in which it induced an increase in cardiac fibrosis and profibrotic factors, resulting in impaired cardiac function. IL-11 is elevated in several models of cardiac fibrosis selected in this study and positively influences profibrotic action, with action dependent on ERK signaling. Thus, IL-11 stands out as a promising therapeutic target for cardiac fibrosis; based on the different interventions investigated, its inhibition proved to be favorable for reversing the harmful effects of tissue remodeling.

## Data Availability

The original contributions presented in this study are included in the article. Further inquiries can be directed to the corresponding authors.
